# Successful Treatment of a Patient With Chronic Bronchiectasis Using an Induced Native Phage Cocktail: A Case Report

**DOI:** 10.7759/cureus.77681

**Published:** 2025-01-19

**Authors:** David A Jernigan, Roman D Hentish

**Affiliations:** 1 Bioregulatory Medicine, Biologix Center for Optimum Health, Franklin, USA; 2 Bioregulatory Medicine, Chronic Illness, Biologix Center for Optimum Health, Franklin, USA

**Keywords:** aspergillus, asthma, biofilm, bronchiectasis, endotoxin, induced native phage therapy, multi-microbial activation syndrome, mycobacterium, pseudomonas aeruginosa, staphylococcus

## Abstract

Bronchiectasis is a well-recognized chronic respiratory disease characterized by a productive cough and multi-microbial activation syndrome (MMAS) of various respiratory infections due to what can be the permanent dilatation of the bronchi. Bronchiectasis represents an ongoing challenge to conventional antibiotic treatment as the damaged bronchial environment remains conducive to ongoing opportunistic infections and microbial mutations, leading to multi-drug resistance.

Standard treatment guidelines are designed to promptly identify and address the primary infection. Despite the strong focus on identification of the primary infection in each new episode, by combining clinical history, and high-resolution computed tomography (HRCT), a high proportion of patients remain classified as "idiopathic". Important underlying infections, such as *Aspergillus *and other mold infections, *Pseudomonas aeruginosa*, *Mycobacterium*, *Mycoplasma*, and various viruses, are frequently not identified for prolonged periods of time, and selected broad-spectrum antibiotics are often ineffective. The introduction of Induced Native Phage Therapy in 2021 and Induced Native Phage cocktails in 2024 provides a new treatment alternative that induces naturally occurring phages to eliminate specifically targeted acute and chronic mixed infections even in cases of multi-drug resistant infections as seen in chronic bronchiectasis.

This article will present the successful long-term results in a case study demonstrating the speed, gentleness, and effectiveness of induced native phage cocktails in a 45-year-old male with life-long asthma resulting in multi-microbial activation syndrome in severe non-cystic fibrosis bronchiectasis for the last 20 years.

## Introduction

According to the World Health Organization, respiratory tract infections have the highest mortality rate and are the fourth leading cause of death of all communicable diseases [1]. Chronic bronchial asthma often leads to the permanent dilation of the bronchi, predisposing the individual to bronchiectasis, which is considered a permanent and irreversible condition. Bronchiectasis is a cause of significant morbidity and mortality. The disease is complicated by multiple drug-resistant infections due to the necessity of prolonged use of antibiotics, steroids, and a weakened immune system, as well as iatrogenic damage to the microbiome diversity, and damage to global cellular mitochondrial function [2]. Systemic bioregulatory instability caused by these and other adverse events leaves the body with no ability to regulate the pathogenesis of microbes and their biofilms. Efforts to upregulate the immune system often only result in a hyperreactive immune system and a dramatic increase in proinflammatory cytokines, making matters much worse. Ongoing environmental exposure to a wide range of infectious microbes, allergens, and toxins create a very challenging condition to treat.

The difficulty of treating chronic bronchiectasis is representative of many other upper and lower respiratory tract infections of mixed origin. The present antibiotic treatment of bronchiectasis is often unsuccessful, leading to the dependency of nearly constant antibiotics over the course of many years. Rapid resolution is almost unheard of in conventional treatment. The conventional understanding is that bronchiectasis is a progressive disorder with no cure, which can result in considerable morbidity and early mortality [3]. Conventional phage therapy, even though frequently successful, usually has two to four wild-sourced phages that are introduced into the body and can only target one infection at a time [4,5]. This case report highlights a novel treatment inducing native monovalent or polyvalent phages in a highly precise broad-spectrum cocktail to target and eliminate 35 potentially pathogenic microbes commonly seen in cases of chronic respiratory infections such as bronchiectasis [6].

## Case presentation

This case study presents the positive impact of Induced Native Phage cocktail, Inducen-Res™ (PhagenCorp, Franklin, TN, USA), in the treatment of a 45-year-old male who developed asthma at the age of two leading to the eventual development of severe bronchiectasis. The patient required ongoing treatment with inhalers, steroids, and antibiotics throughout his life. In 2004, his bronchial condition worsened for unknown reasons, with the subsequent X-ray diagnosis of a collapsed upper right lobe, which reinflated with prednisone treatment. Scarring in the lungs was also noted. It was at this time that the diagnosis of bronchiectasis was made. Monitoring with annual CT scans has been ongoing until entering the study in April of 2024. The patient reported he had no lifestyle aggravating factors such as smoking or recreational drug use. He reported no history of sinusitis, dental infections, or issues with the digestive tract. No hilar lymphadenopathy was reported historically. Crackles were noted upon auscultation periodically over the last 20 years in association with bouts of infections, along with excessive coughing, production of yellow-brown mucus, and continuous dyspnea without inhalers. The patient reports having to take various antibiotics three days per week for many years. Infection returned within two to four weeks of every attempt to discontinue the antibiotic therapy over the 20-year duration.

Treatment and symptom improvement

The direct treatment agent for the multiple infection presentation was Inducen-Res™, a prepared liquid oral solution imprinted with the induction signatures required to induce diverse populations of native monovalent and polyvalent phages to eliminate each of the microbes addressed within the formulation. Dosages were self-administered in individually dosed 2 ml sterile oral solution in glass ampules. The daily dosage recommendation, to which the patient was compliant, was two ampules three times daily orally, swish and swallow in a naturally clean mouth for seven days with reduction to one ampule twice daily (a.m./p.m) for six weeks. Though the duration was initially set for six weeks, this dosage was maintained for six months at the patient’s request to remain on the treatment for fear of a return of the infection, in spite of dramatic improvements experienced by the patient. The patient was monitored remotely during the follow-up stage of care. Repeated doses are necessary to keep the native phages induced to continue in their lytic state against the targeted infections, a very unnatural condition for most phage types. The induction process does not induce all the phages of the body to turn lytic. Only types of native phages that can target the type of infections represented by the formulation are activated. No other antimicrobial treatments were utilized.

During the first two weeks the patient underwent a comprehensive program of outpatient treatments designed to facilitate the restoration of the optimal structural and functional integrity of the body and to restore coherence within the bioregulatory aspects of the body. Daily supportive therapies included parenteral nutrition consisting of magnesium, high-dose thiamine, B complex, vitamin C, methylcobalamin (B12), and glutathione. Other therapies included lymphatic drainage therapies and therapies to support improvement of microcirculation and rapid detoxification, as well as therapeutic massages.

The patient entered the treatment program in April of 2024, with his collective symptoms of bronchiectasis reported at an 8/10 severity. These symptoms included but were not limited to dyspnea, wheezing, crackles, excessive mucus, mucus plugs and coughing. He reported persistent flu feelings with fatigue. He was examined and was found to have objective neuromuscular and musculoskeletal deficits with associated infections as determined by adjunctive testing and case history and presentation. Treatment began the following day. Three days after initiating treatment, his symptoms were reported as a 4/10 at which point, he was no longer coughing up mucus, but remained on his antibiotics, inhaler and continued with the 15 mg of prednisone as prescribed by his primary physician. After just three days of treatment with the Inducen-Res™, he went off his antibiotics by his own choice. His improvements continued and culminated to a reported 0/10 with no relapses through to September 2024, with continuous twice-daily, 2 ml doses of Inducen-Res™. The patient returned for re-evaluation, six months from beginning treatment, in September, 2024. Upon reexamination no evidence of infections could be found. Patient was instructed to discontinue treatment with the Inducen-Res™ at that time.

Notably, the patient no longer required his asthma inhaler and voluntarily discontinued use. Upon follow-up consultation approximately nine months after the initiation of treatment in April and three months after discontinuing the Inducen-Res™, there has been no recurrence of infection, no coughing, mucus, or dyspnea/wheezing. Physician-guided gradual reduction of the prednisone has brought the dosage down to 8 mg daily with the goal of complete elimination of this medication.

To evaluate the patient’s post-treatment progress, a series of detailed follow-up assessments were conducted with a verbal short-form questioning for quality of life. Table 1 illustrates the significant improvements in overall quality of life.

**Table 1:** Improvement in quality of life symptoms before and after treatment SF-36: 36-Item Short Form Health Survey, FSS: Fatigue Severity Scale, VAS: visual analog scale

Parameter	Pre-treatment	Post-treatment	Interpretation
General health (SF-36)	40%	85%	Significant improvement in overall well-being and physical health.
Energy (SF-36)	50%	85%	Substantial increase in energy levels indicates enhanced physical and mental stamina.
Social (SF-36)	50%	85%	Improved social functioning reflects better social interactions.
Health Change (SF-36)	40%	90%	Improvement in perceived health change shows a positive perception of health progress.
Fatigue (FSS Score)	1-50%	50-80%	Fatigue severity decreased from high to low, demonstrating effective alleviation of fatigue.
Pain (VAS Score)	Flu-like Achy	None	Pain severity reduction from severe to moderate demonstrates the efficacy of the treatment.
Cognitive Function	50%	70%	Cognitive function improved from low to acceptable range.
Need for Pharmaceutical Treatments	100%	0%	Substantial overall improvements.

## Discussion

In this case study, a standardized Induced Native Phage cocktail formulation, called Inducen-Res™ was given to the patient in multiple doses over a seven-day period, with follow-up doses as needed. Follow-up dosing was determined by respiratory status and bioregulatory outcome measures. This strategy was designed to address the multi-microbial activation syndrome (MMAS), broad-spectrum respiratory infections and coinfections, as well as induced native phage therapy (INPT) signatures targeting native phages to eliminate bacterial toxins associated with his treatment-resistant non-cystic fibrosis bronchiectasis.

The following lists the microbes which were specifically targeted in the standardized formulation Inducen-Res™ used to treat this patient: Bordetella holmesii, Bordetella pertussis, Burkholderia cepacian + lipopolysaccharide (LPS)/endotoxin, Burkholderia pseudomallei + endotoxin/LPS, Chlamydophila pneumoniae (Chlamydia pneumoniae), Haemophilus influenzae, Klebsiella pneumoniae, Legionella pneumophila, Moraxella catarrhalis, Mycoplasma pneumoniae, Mycoplasma hominis, Mycoplasma fermentans incognitus, Mycobacteria Avium complex, Mycobacterium abcessus, Mycobacterium kansasii, Mycobacterium intracellularis, Mycobacterium tuberculosis, Mycobacterium tuberculosis (multi drug-resistant), LPS + endo/exotoxins, Pneumocystis carnii, Prevotella melaninogenica Pseudomonas aeruginosa, Pseudomonas fluorescens, Serratia marcescens, Methicillin-resistant Staphylococcus aureus (MRSA), Staphylococcus aureus, Streptococcus pneumoniae, Streptococcus pyogenes, Streptococcus fecalai, Streptococcus moniliformis, Streptococcus mutans, Streptococcus agalaciae, Stenotrophmonas maltophilia, Candida albicans, Aspergillus fumigatus, Aspergillus niger, Coccidioides sp., and Protomyxzoa rheumatica.

Each strain and species was specifically and individually targeted with the INPT signatures within the INPT cocktail, Inducen-Res™. It is impossible to induce a native phage to target an infection or strain the person does not have. Native phage cocktails have been developed that can target over 100 types of infections which are typically or atypically seen in each disease process being addressed by the various Inducen formulations. Although a patient may not have a hundred types of problematic infections, and may only have one or two types, only the types of infections they do have will be relevant from those included in the INPT cocktail. The extraneous induction-signatures appear to dissipate harmlessly. Prior to the development of induced native phage cocktails, the targeting of the primary infection, when effective, left a vacancy for opportunistic infections to rise to the level of priority, especially when the internal mileau had not been improved. In that the terrain of the body is not being addressed by the Inducen formulation, other treatments and therapies to improve bioregulatory aspects of the body are needed to avoid opportunistic infections from taking dominance in the absence of the now-eliminated infections.

Not only can the specific numerous primary infections and coinfections be targeted with INPT cocktails, but also the microbial toxins, such as endotoxins, exotoxins, and LPS can be targeted at the same time. Many of the targeted microbes are known to produce formidable biofilms, however fluorescing microscopy studies have verified the effectiveness of phages dissolving these biofilms [7].

Henrikson et al. demonstrated that even when conventional phage therapy was only partially successful, it still decreased biofilms and increased the effectiveness of more classic antibacterial treatments [8]. It is hypothesized that the induction of native phages to target microbial toxins such as the lipopolysaccharide bacterial toxins from within the biofilms can be accomplished on location. The goal of inducing native phages to eliminate the infections rapidly, as well as degrade the microbial toxins appears to be effective based upon favorable subjective clinical results [9]. The concern that inducing specific native phages to eliminate the targeted infections might inadvertently activate bacterial pathogenicity via increased production of toxins, has not been observed in the four years of treatment of hundreds of study participants. Over 150,000 oral doses have been given under IRB-supported research protocols for a wide range of conditions with no reported adverse effects and absent to minimally apparent Jarisch-Herxheimer (JH) reactions when combined with comprehensive therapies even with microbes known typically to illicit strong JH reactions.

It is important to note that native and wild conventional phages can penetrate microbial biofilms to reach the infections. Microbial biofilms often contain the primary substances of pathogenicity beyond the actual microbial infection, so any pharmaceutical treatment that targets only the infection may result in moderate symptomatic relief and yet not change the underlying disease process due to the residual biofilms [10].

Given the complexities and challenges of diagnosing and treating acute and chronic multi-microbial activation illnesses such as bronchiectasis, our decision to treat using a broad-spectrum, precision-targeted induced native phage cocktail was justified by several factors. Previous antibiotic and steroidal treatments had failed to completely reduce the symptoms. The patient believed there were diminishing returns with the pharmaceutical treatment, feeling he was experiencing a gradual decline in his overall quality of life. Inducen-Res™ had four years of successful use in clinical studies for a wide range of clinical symptoms of respiratory tract infections. Additionally, the patient had attempted numerous types of natural medications without effect.

Despite not having a primary diagnosis of the specific types of infections involved in total, previous culturing of various known infections, such as Aspergillus, Pseudomonas aeruginosa, and others, the typical and atypical opportunistic infections could be anticipated. The broad-spectrum but precision-targeted native phage induction of the Inducen-Res™ formulation was justified due to the nature of the poor condition of the bronchi creating a diverse microbial community. Targeting only the worst infections would leave a vacancy for other microbes to take dominance.

In retrospect, maintaining constant pressure to induce the native phages with Inducen-Res™ for six months, as opposed to discontinuing the treatment after the initial six weeks as expected, likely enabled the damage to the bronchial tissues from the years of chronic inflammation to heal without the hindrance of newly acquired infectious agents. Inducing native phages as in INPT is a manipulation of only those types of native phages that can and will eliminate the targeted infections. As this induction is not part of their natural function of lysogeny, pressure must be maintained to keep them virulent against the targeted infections. It does not appear that native phages will stay induced upon discontinuation of the induction process, nor is there any apparent added protection against future infections. 

## Conclusions

This case highlights the effectiveness of the new treatment technology, Induced Native Phage Therapy and Induced Native Phage cocktails in addressing illnesses with acute or chronic diverse infections. The apparent speed and effectiveness of clearing multiple infections, the observed ease of tolerance of the treatment, and the apparent sustained elimination of the infections are all hallmarks of any successful phage therapy, whether conventional or induced native phage therapy. Inducen™ formulations are still in the clinical research phase. The dramatic success of this case underscores the value of this new treatment modality that harnesses the benevolent and beneficial native phages that inhabit the human body. It is understood that patient compliance is critical to maintaining pressure on the native phages to rapidly achieve their goal of eliminating the total targeted microbial populations before those populations can develop phage resistance.

Induced Native Phage cocktails safely and cost-effectively offer precision targeting of multiple infectious agents within the same treatment, as opposed to conventional (wild) phage therapy, which typically only addresses a single infectious agent. Contrasted with conventional phage therapy, which most often requires the identification of the primary infection being matched with a cocktail of externally sourced phages, the ease of customization of Induced Native Phage cocktails to target diverse infections enables rapid deployment to the physician to provide a treatment when the exact infection is known or without knowing exactly which infectious agents are causing a patient's illness. It is recognized that more studies need to be performed on patients with bronchiectasis and other respiratory infections, as well as with the various illness-specific induced native phage cocktails that have already been formulated for other MMAS illnesses, such as fungal infections, urinary tract infections, Lyme disease and relapsing fever infections, gastrointestinal infections, and neurological infections.
